# Mobility restriction and barrier-reduced housing among people aged 65 or older in Germany: Do those who need it live in barrier-reduced residences?

**DOI:** 10.3389/fpubh.2023.1098005

**Published:** 2023-04-21

**Authors:** Sonja Nowossadeck, Laura Romeu Gordo, Alberto Lozano Alcántara

**Affiliations:** German Centre of Gerontology, Berlin, Germany

**Keywords:** barrier-reduced housing, mobility restriction, aging in place, moving in old age, Germany

## Abstract

**Introduction:**

Older people spend a lot of time at home and in the area near where they live. Housing conditions ensure their ability to participate in social life, especially when they suffer from mobility restrictions. Barrier-free access to the residence and to rooms within the residence is a key condition for their everyday mobility. As a result, this is what we define as minimal criteria for barrier-reduced residences. This article examines the extent to which people aged 65 and over (including people with mobility issues) live in barrier-reduced housing and what factors influence the chance of living in such residences.

**Data and method:**

Cross-sectional data from the German Ageing Survey (DEAS) 2020/21 (persons aged 65 and over, *n* = 2,854) were used. The DEAS is a representative cross-sectional and longitudinal survey of the population aged 40 and over in Germany. In our analyses, we used logistic regression models to investigate the probability of living in a barrier-reduced residence. We defined housing as barrier-reduced when the apartment/house and the rooms inside it can be reached without steps or stairs. As explanatory variable, we considered mobility restrictions, defined as limited ability to climb a flight of stairs. In addition, the model includes other individual factors (age, gender, equivalized household income), regional factors (living in East vs. West Germany, in urban vs. rural region) and moving to the current residence after the age of 65.

**Results and discussion:**

Of all individuals aged 65 or older, 19.3 percent live in a barrier-reduced residence. Also, of mobility-restricted elders, only 21.4 percent have such residences. The logistic regression results show that mobility restrictions are associated with a higher probability of living in a barrier-reduced residence. Compared to the lowest income group, older people in the highest income group are more likely to live in barrier-reduced housing. East Germans and people in urban areas are less likely to live in a barrier-reduced home. The likelihood of barrier-reduced living is higher among seniors who moved into their current residence after age 65. No significant differences were found for age groups and gender. The findings show that not enough seniors have barrier-reduced access to their homes and rooms, even if they suffer from mobility restrictions. Preventing functional restrictions must therefore also include improvements in the residential environment, especially in disadvantaged residential areas.

## 1. Introduction

Most people live in their own private household into their old age. Even though the variety of living arrangements in old age has increased in recent years, with assisted living and shared apartments, for the majority of older people living in their own, private household remains the desired form of living and also the reality they live in. Even if seniors are functionally limited or require care, they remain in their homes (1). Aging in place is the main preference both in German households and in other European countries (2). The home is increasingly becoming the center of life in old age. With increasing age and health problems, older people reduce their radius of action and spend more time in their homes and the immediate area (3). At the same time, their vulnerability to deficiencies in the home and the living environment increases. For older people, the home and its environment therefore determine their level of self-determination as they age to a significant extent (4).

Barrier-free housing is one aspect of housing quality for people of all ages and in all circumstances of life. It also makes daily life easier for families with children or younger people with functional limitations. Therefore, barrier-free living is not limited to old age, though it is especially significant for this phase of life because of the frequent age-related health problems. With an aging population and the resulting increase in the proportion of older people, the need for housing adapted to their specific needs is growing. It should at least be available to mobility-restricted older people to enable them to live independently.

How did we define barrier-free housing for our analyses? In Germany, there is no uniform, generally binding definition of barrier-free living. There are different target groups with different accessibility needs and different places and spaces with varying possibilities for intervention (5). This is reflected in a variety of legal regulations and building standards on accessibility.

When it came to defining accessibility, we considered a range of factors. Older people are often limited in their ability to climb stairs. Even the aids they need for mobility (e.g., walkers) can be a hurdle if they have to be transported up multiple flights of stairs. Freedom of movement within the home is similar to access to the home. Steps and higher thresholds are potential trip and fall hazards, and they make it difficult to move with a rollator within the home. For our study, accessibility is therefore defined according to two criteria related to the accessibility of the residence and the rooms. This can only be considered as a minimum standard, so we do not speak of barrier-free residences in the following, but rather of barrier-reduced residences. Our definition of barrier-reduced residences is limited to aspects that are essential for everyday mobility, especially for elderly with mobility restrictions: step-free access to the dwelling and step-free access to all rooms in the dwelling (see Data and methodology section). This definition of accessibility, which can be measured well with survey data, provides a good overview of the situation of barrier-free housing by minimum criteria.

In this context, it is relevant to investigate who is more likely to live in barrier-reduced housing after the age of 65 and whether people who are more in need of barrier-reduced living conditions do actually have them. Further, in this paper we investigate the role of household income in determining adequate housing for groups with special needs. In particular, we want to address the following research questions:

1. How many of those aged 65 and over live in barrier-reduced housing, i.e., how many have barrier-free access to the residence and the rooms inside it? Is there a matching of need and conditions, i.e., do people with special needs (people with mobility limitations) live in “suitable” housing?
2. What factors influence barrier-reduced housing conditions in older age? Is income a determinant of barrier-reduced housing? Are people with more needs (people with mobility restrictions) with low income levels less likely to live in adequate housing than people with higher income?

## 2. Data and methodology

The analyses were conducted using data from the German Ageing Survey (DEAS), a representative cross-sectional and longitudinal survey of individuals in the second half of life (6). We used data from the 2020/21 survey, which took place from November 4, 2020, to March 1, 2021. A total of 5,402 individuals aged 46 and over participated in the survey. All respondents had participated in the survey at least once before. Due to the COVID-19 pandemic, individuals were interviewed by telephone. Following the telephone interview, respondents were sent a questionnaire that could be answered in writing or online. As we want to focus on those who spend more time at home and in the neighboring area, and those who have higher probabilities of having mobility restrictions, only individuals aged 65 or over living in private households were included in our analyses (*n* = 2,854).

To compensate for the disproportionate sampling, data weighting was applied (7). For this purpose, marginal adjustments of the sample were made to the relative frequency of the characteristic combinations of the sample stratification of age group, sex, and part of the country in the official population statistics. The weighting factors are used for the univariate and bivariate representations.

Accessibility: In our analyses, we used the accessibility of the residence as a dependent variable. Information on the accessibility of the residence was requested in the written questionnaire – respondents were asked to assess features of their residence, such as access to the residence, accessibility of rooms, and other characteristics. The total set of potential variables was not used to define the accessibility of the residence. The proportion of respondents living in an accessible residence according to all criteria recorded in the questionnaire is very small. In 2014, according to DEAS data, it was only 2.9 percent of people aged 40–85 and 5.6 percent of those aged 70–85 (4). Therefore, in order to have a larger sample size available for the analyses on accessibility, the dependent variable was defined as a barrier-reduced residence, based on minimal criteria. Barrier-reduced housing in this sense is coded as 1 when the respondents answered positively that their “apartment or house is accessible without steps” and that “within the apartment or house, all rooms are accessible without steps,” and is coded as 0 when they answered negatively. The following characteristics are included as explanatory variables (see Table 1):

**Table 1:** Sample characteristics of participants (*n*, %).

	*n*	%
**Lives in barrier-reduced residence**		
No	2,263	80.7
Yes	540	19.3
**Mobility restriction**		
Not restricted	2,132	74.8
(Severely) Restricted	718	25.2
**Age group**		
65–79	1,925	67.4
80 +	929	32.6
**Gender**		
Male	1,294	45.3
Female	1,560	54.7
**Equivalized household income**		
Quintile 1 - lowest	683	24.9
Quintile 2	524	19.1
Quintile 3	500	18.2
Quintile 4	558	20.3
Quintile 5 - highest	480	17.5
**Region**		
West Germany	2,272	79.6
East Germany	582	20.4
**Regional typology**		
Rural	1,025	35.9
Urban	1,829	64.1
**Moving after age 65**		
No	2,435	85.3
Yes	419	14.7

Source: DEAS 2020/21. Weighted number of cases and weighted frequencies.

Mobility restriction: As described above, we use minimal criteria of accessibility, which include barrier-free access to the residence and to the rooms in the residence. Therefore, it seems reasonable to measure functional limitations of the respondents in daily life with barriers at the residence. For this purpose, we measure respondents’ mobility restriction with item 5 (“Climbing a flight of stairs”) from the subscale “Physical Functioning” of the 36-item short-form health survey (SF-36) (8, 9): “The following questions are about activities you might do during a typical day. Does your health now limit you in these activities? Are you severely restricted, somewhat restricted or not restricted due to your current state of health?” (10). We code in 1 = “severely restricted” or “somewhat restricted,” and 0 = “not restricted.”

Age groups: We differentiate age groups of 65–79 and 80 years and older.

Gender: We differentiate male and female persons.

Equivalized household income: This variable contains the needs-adjusted net monthly per capita income of the household. Weighting of household size uses the modified OECD equivalent scale that is used by Eurostat and the Federal statistical Office (10). This information is introduced in the form of quintiles.

Region: We differentiate between West Germany and East Germany.

Regional typology: We differentiate rural and urban areas of living, and use information on the urban–rural type of district based on structural characteristics of the settlements (see (10)). Four district types are defined: “metropolitan districts,” “urban districts” (both combined and coded by us as “urban”), “(partially) densely populated rural districts” and “sparsely populated rural districts” (both combined and coded by us as “rural”).

Moving after age 65: We use the information from the questionnaire about how long the respondent has lived in the current residence and their age to calculate whether or not the person moved into this residence after age 65.

Table 1 shows that 19.3 percent of individuals aged 65 or older in Germany live in barrier-reduced housing. That is, only about one in five at this age can get into the dwelling and rooms inside it without having to climb steps. Some 80.7 percent therefore do not have barrier-free access to their dwelling and rooms.

Almost 33 percent are 80 years old or above, and about 20 percent live in East Germany. Most individuals live in an urban area (64 percent). Finally, only around 15 percent moved house after age 65, which confirms the preference of aging in place.

The probability of living in a barrier-reduced residence is estimated using multivariate logistic regression analysis with a binary dependent variable (Respondent lives in a barrier-reduced dwelling yes/no).

## 3. Results

### 3.1. Descriptive results

First, we bivariately examined the distribution of over-65 s living in barrier-reduced residences by sociodemographic and regional variables for those individuals with and without mobility restrictions.

**Figure 1:**
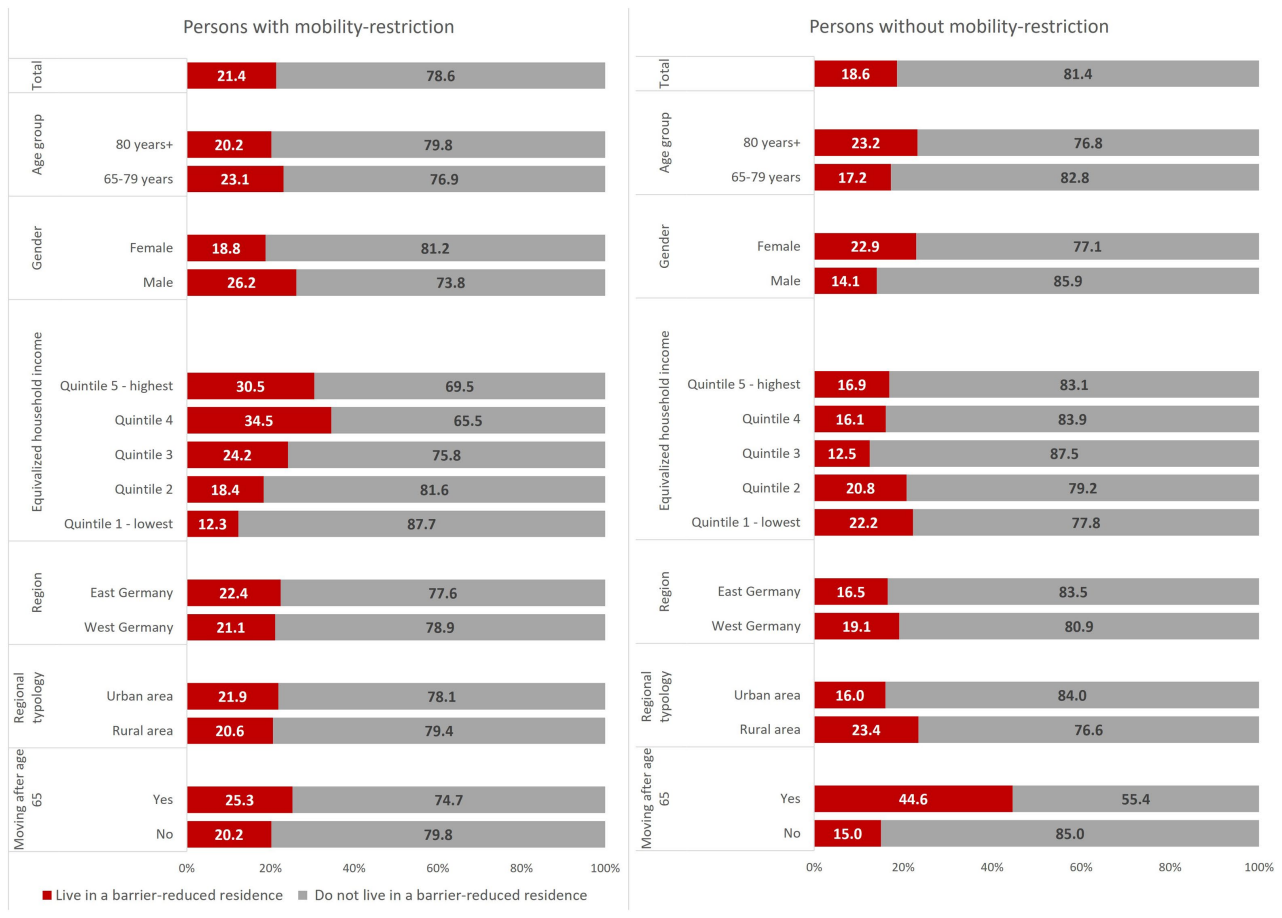
Proportions of persons with or without mobility-restriction who (do not) live in a barrier-reduced residence (%). Source: DEAS 2020/21 (*n* = 2,800). Weighted frequencies.

What Figure 1 shows is that only a small percentage of people – both with and without mobility problems – live in barrier-reduced residences (21.4 and 18.6 percent respectively). However, differences between the two groups are small, meaning that the overall level of barrier-reduced housing in Germany is low. In addition, considering that older people who are still mobile at present may also develop limitations in the course of the next few years, the need for barrier-reduced housing will increase.

Men with mobility restrictions more often (26 percent) live in suitable housing than women with mobility restrictions (19 percent). We do not observe large differences between urban and rural areas for people with mobility restrictions, but we do see differences in the case of people without mobility restrictions. People living in rural areas more often live in a barrier-reduced residence than those in urban areas.

Income seems to be relevant for the accessibility of appropriate housing for people with mobility restrictions. We observe in Figure 1 that people in the upper income quintile more often live in barrier-reduced houses than people in the middle or lower quintiles. About 31 percent of people in the fifth income quintile (highest incomes) with mobility restrictions live in an adequate residence, while this is only the case for 12 percent of people in the poorest quintile. We do not observe this strong income effect on the group of people without mobility restrictions.

Finally, we observe that people who moved after the age of 65 more often live in barrier-reduced residences. This is particularly the case in the group of people without mobility restrictions. In this case, 45 percent of the individuals who moved late in life live in a barrier-reduced home, while this is only the case for 15 percent of those who had not moved house. We also observe this effect among people with mobility restrictions, but to a lesser degree. While 25 percent of those who moved in later life live in an adequate home, this is only the case for 20 percent of those who had not moved.

### 3.2. Multivariate results

By means of a multivariate logistic regression analysis (see Table 2), we examined how individual characteristics are related to living in a barrier-reduced residence. These relationships can provide initial indications of which factors influence barrier-reduced housing in older age. In a stepwise model, we successively introduced mobility restrictions, sociodemographic factors, and regional factors, or the variable indicating whether someone has moved in old age, into the analysis.

**Table 2:** Determinants of living in a barrier-reduced residence among those 65 or older.

	Model 1	Model 2	Model 3
	Marginal Effects		
**Mobility restriction (ref. not restricted)**			
(Severely) Restricted	0.082^***^	0.071^***^	0.058^***^
**Age group (ref. 65–79)**			
80 +		0.045^***^	0.013
**Gender (ref. male)**			
Female		−0.020	−0.021
**Equivalized household income (ref. Quintile 1 - lowest)**			
Quintile 2		0.032	0.029
Quintile 3		0.030	0.025
Quintile 4		0.033	0.029
Quintile 5 - highest		0.074^***^	0.058^***^
**Region (ref. West Germany)**			
East Germany			−0.044^***^
**Regional typology (ref. rural)**			
Urban			−0.032^**^
**Moving after age 65 (ref. no)**			
Yes			0.232^***^
*Pseudo-R* ^2^	0.0076	0.0145	0.0583
Observations	2,832	2,731	2,731

Source: DEAS 2020/21 (n = 2,832 − 2,731). Binary logit regression analysis. Dependent variable: Living in a barrier-reduced residence (0 = no, 1 = yes). ***p < 0.01 (significance). **p < 0.05 (significance).

In the first model, only mobility impairments are considered. The probability of mobility-restricted seniors living in a barrier-reduced residence is 8.2 percentage points higher than for seniors without mobility restrictions, so this health condition is an important factor in barrier-reduced housing.

Sociodemographic variables (age, gender, household income) are included in the second model. In comparison to those aged 65–79 years, the probability of living in barrier-reduced housing is 4.5 percentage points higher among people over 80. Older people who belong to the quintile with the highest income are 7.4 percentage points more likely to live in such a dwelling than the people in the poorest quintile. Controlled for age, gender, and household income, the proportion of mobility-restricted seniors living in barrier-reduced housing is 7.1 percentage points higher than in those without mobility restrictions.

In a last step (model 3), region, regional typology and the variable indicating whether the individuals had moved after the age of 65 are included in the analysis. The results of this model are:

Mobility restriction: A mobility restriction, measured by the restricted ability to climb a flight of stairs, has a positive effect on the likelihood of living in a barrier-reduced residence. Respondents who can manage a flight of stairs only with restrictions or even severe restrictions have a 5.8 percentage point higher proportion of barrier-reduced housing.

Sociodemographic characteristics: Belonging to the oldest age group (over 80s) has no significant statistical effect on the probability of living in a barrier-reduced residence after controlling for the other characteristics considered in the model. This initially surprising finding suggests that the very old are not more likely to live in such residences than the less elderly. This means that very old age is not necessarily linked to barrier-reduced housing, but other factors, which are closely linked to old age, favor such housing.

Gender also has no statistically significant relationship to barrier-reduced housing in our results. This result might be explained by the fact that a large proportion of those over 65 live together as a couple in the same residence, making it difficult to isolate the gender effect.

There is some evidence in the literature that the economic situation of seniors may also influence how often they live in barrier-reduced housing conditions. To measure the economic situation of the respondents, we used the equivalized household income in quintiles as an indicator. After controlling for other variables, our results show a statistically significant effect of the income quintiles on the prevalence of barrier-reduced housing. Compared to the lowest income quintile, respondents in the highest income quintile have a 5.8 percentage point higher chance of living in barrier-reduced housing. However, we do not observe this effect for the other income quintiles. Only large income differences seem to influence the chance of barrier-reduced housing.

Region and regional typology: Respondents in East Germany are 4.4 percentage points less likely to live in a barrier-reduced residence than respondents in West Germany, even after controlling for mobility restrictions and sociodemographic variables. Living in urban areas reduces the chance of barrier-reduced housing by 3.2 percentage points compared to living in a rural area.

Moving after age 65: Moving after age 65 has a large positive effect on the likelihood of living in a barrier-reduced apartment. Those who moved to their current home after age 65 are 23.3 percentage points more likely to live in barrier-reduced housing than seniors who did not.

We also included the interaction effect between quintile of household income and mobility restrictions in the model to test whether people with higher incomes and restricted mobility are more likely to live in barrier-reduced housing than people with lower incomes and restricted mobility. In our model (results are not shown), such an interaction has no significant effect. Further we have tested the assumption that higher-income seniors are more likely to move at older ages than low-income seniors. This interaction between moves and income was also not significant in the model.

## 4. Discussion

### 4.1. Most older and mobility-restricted people in Germany do not live in barrier-reduced housing

We directed our analyses to barrier-reduced housing for individuals aged 65 or older in Germany, with a special focus on people with mobility restrictions. One main finding is that there is not enough barrier-reduced housing. This is true even based on our minimum criteria, which only include barrier-free access to the residence and the rooms within it. Only 19.3 percent of all over-65 s live in barrier-reduced conditions, meaning over 80 percent do not. Even among the very old over 80, only 21.3 percent are provided with barrier-reduced residences. This percentage can be assessed as very low, considering the importance of mobility in the residence and in its surrounding area for this age group. Even more serious is that only 21.3 percent of those aged 65 or older with difficulties climbing stairs live in a barrier-reduced residence. Our findings are consistent with earlier studies that showed only about 3 percent of all 40 to 85-year-olds had barrier-free housing (4). Barrier reduction in the home is not the only prerequisite for successful aging in place. As another essential aspect of housing in old age, technical support and its acceptance should be mentioned here (11).

### 4.2. Advanced age alone is not a key indicator for barrier-reduced housing – mobility restrictions have a significant impact

The results of our multivariate analysis show that, when controlled for other characteristics, advanced age of over 80 years does not determine whether seniors live in barrier-reduced housing or not. Other variables such as the existence of health problems, measured as whether someone suffers mild or severe mobility restrictions, increase the probability of living in barrier-reduced housing (by 5.8 percentage points). This implies that people with more needs are more likely to live in appropriate housing. However, as the descriptive results show, the percentage is still very low. According to the descriptive results, individuals aged 80 or over are also more likely to live in barrier-reduced housing than the younger age group of individuals aged between 65 and 79. However, the moment we also control for mobility problems, the effect is not statistically significant anymore, as age and mobility restrictions are highly correlated.

### 4.3. High income is positively related to barrier-reduced housing

We could see in the bivariate analyses that mobility-impaired seniors in the higher income quintiles were more likely to live in barrier-reduced housing than those in the lower quintile. We also see in the multivariate model that people with high incomes live more often in barrier-reduced houses, but only in comparison to the lowest income quintile. Factors influencing barrier-reduced housing such as moving may overlay the income effect in the middle income groups. Such factors may also be socially unevenly distributed, but we cannot measure this in the model.

### 4.4. Less barrier-reduced housing in East Germany

In addition, our results show that regional characteristics also play a role. Older people in East Germany are less likely to live in barrier-reduced housing than older people in West Germany. It seems that there is less availability of barrier-reduced housing in East rather than West Germany. With these regional differences, it can be assumed that the income differences between East and West Germany play a role. It can also be assumed that residential buildings in East Germany are older on average than in West Germany and that this fact favors differences in barrier-free living. Other differences between both regions such as lower homeownership rates in East Germany or differences in the structural types of houses may also explain such differences.

### 4.5. Those moving in older age could have an advantage in barrier-reduced housing

Our analyses also show that moving in old age is correlated with barrier-reduced housing in old age. In these cases, there is a high probability that housing is adapted to the needs elderly people have when they move. This finding suggests that people moving at older ages are often motivated by changing to more appropriate (in terms of accessibility) housing. Indeed, moving after age 65 has the strongest impact on the likelihood of living in a barrier-reduced residence.

Our findings are consistent with previous findings that proved the role of long periods of residence in old age. Höpflinger (12) speaks of double aging in the case of a long period of residence – the aging of the people themselves and the aging of their home. He notes that, with a long period of residence, the dwelling and neighborhood take on a high affective significance. Therefore, a long period of residence can go hand in hand with a high level of residential satisfaction due to habituation, even in the case of housing that is not suitable for seniors.

In Germany, there are very long periods of residence and little residential mobility. It can be assumed that people in middle adulthood who are looking for a new home do not select it primarily according to the criterion of accessibility. As they grow older and become familiar with the living arrangements, neighborhood, and environment, there is little incentive to move to another, possibly barrier-reduced, residence. It is only as functional health deteriorates that barriers in the residence can become a real obstacle to daily life. By then, however, the burden of moving or conducting extensive construction work in the residence will have become disproportionately high.

What strengths and limitations do we identify in our study?

One strength is that a set of housing characteristics are collected for a representative population sample, which are necessary for the formation of the barrier-reduced housing indicator. Limitations lie in the fact that we used a panel sample for our cross-sectional analyses. Weighting factors were used to compensate for bias within the sample. The housing information is self-reported by the respondents, so the assessment of barriers is subjective and not based on objective metrics or measurements. Our results are correlations and do not show any causal effects. In addition, it must be remembered that the 2020/21 study was conducted during the COVID-19 pandemic, which may have influenced willingness to participate and response behavior.

What can we conclude from our results?

Living in a private household in old age remains a balancing act between individual living wishes, holding onto familiar places and networks, and the objective conditions and possibilities offered by the built environment for carrying out daily tasks and requirements. Aging in place therefore requires that housing is adequate to the special (and changing) needs of the older generation. If this is provided, aging in place is possible and desirable for both seniors and society. Age-appropriate housing requires there to be enough apartments with barrier-free housing standards and that these apartments are affordable to the elderly population. Our results show that the need for age-appropriate housing for the over-65 s is far from being met. An interesting question for future research is how living in inadequate housing conditions affects the probability to living in a nursing home. Barrier-reduced living in old age can be realized by modifying the existing apartment or by moving into an appropriate apartment. Both options require a great deal of financial and organizational effort on the part of the older person. It is therefore necessary to educate older people with housing advice about the options available for age-appropriate housing conversion and financial support. If older people are looking for a new apartment and are willing to bear the burdens of a move, they should be supported in finding an apartment and moving. The shortage of housing in many regions of Germany should not lead to a reduction in age-appropriate housing standards for older people.

With our analyses, we can only depict a small part of age-appropriate living, because in addition to barrier-reduced housing, a barrier-reduced living environment, local availability of essential infrastructure facilities, and social and nursing support services are also part of age-appropriate living (13). The demographic aging of the population makes it particularly necessary to pay more attention to these aspects.

Our results confirm findings from research in other countries on barrier-free housing: A study in five European countries (14) showed for people aged 75 and older, that those who had better accessible homes and who perceive their home as meaningful and useful are more independent in daily activities and have a better sense of well-being. A study from the U.S. (15) examined factors older adults view as barriers to their aging in place plans. The study finds that for elders, barriers and conditions for safety in the home are essential and that elders need better, person-centered informed support to adapt housing conditions to their needs. Another South Korean study (16) revealed that barrier-free housing is an important choice for older people and can be adopted by them as an affordable housing option. The value of barrier-free housing can exceed its cost in this regard. If the willingness to pay of people who demand barrier-free housing is higher than the cost of it, barrier-free construction can be a sustainable marketing option in the housing market.

Policymakers in Germany have recognized that there is too little barrier-free housing and that the need will increase in view of demographic developments. Through the “Kreditanstalt für Wiederaufbau” (Reconstruction Loan Corporation), the German government has initiated a program for the age-appropriate conversion of housing, which provides funding for the removal of barriers in existing buildings (17). Germany is not an exception internationally in terms of barrier-free housing for people with functional limitations. As the OECD states in a study, there is a general lack of accessible housing for people with disabilities in OECD and EU countries. At the same time, financial barriers keep these people from housing conditions that meet their needs, especially since they often live in precarious financial conditions. However, there are also information barriers that make current housing offers and information about corresponding services difficult to reach for potential users (18). The lack of affordable housing, especially in large cities and their agglomerations, is an increasingly serious problem in Germany. There is a danger that affordable barrier-reduced housing will become unattainable for many people with disabilities, not only in old age. This must be counteracted by politics at the federal level, but also by local politics.

## Data availability statement

Publicly available datasets were analyzed in this study. This data can be found here: The datasets presented in this article are available from the Research Data Centre of the German Centre of Gerontology. Requests to access the datasets should be directed to https://www.dza.de/forschung/fdz/kontaktformular.

## Author contributions

SN, LRG, and ALA contributed to conception and design of the study. SN organized the database, performed the statistical analysis, and wrote the abstract and the first draft of the manuscript. LRG and ALA added parts of the sections of the manuscript. All authors contributed to the article and approved the submitted version.

## Conflict of interest

The authors declare that the research was conducted in the absence of any commercial or financial relationships that could be construed as a potential conflict of interest.

## Publisher’s note

All claims expressed in this article are solely those of the authors and do not necessarily represent those of their affiliated organizations, or those of the publisher, the editors and the reviewers. Any product that may be evaluated in this article, or claim that may be made by its manufacturer, is not guaranteed or endorsed by the publisher.
